# Basic Studies on the Oxidative Stress Markers in Two Types of Horse Breed: Semi-isolated Population of Huculs Is Different from Commercially Used Arabian Horses

**DOI:** 10.1155/2020/7542384

**Published:** 2020-07-13

**Authors:** Barbara A. Bażanów, Elżbieta Chełmecka, Ewa Romuk, Dominika M. Stygar

**Affiliations:** ^1^Division of Microbiology, Department of Pathology, Faculty of Veterinary Medicine, Wroclaw University of Environmental and Life Sciences, Wrocław 50-375, Poland; ^2^Department of Statistics, Department of Instrumental Analysis, Faculty of Pharmaceutical Sciences in Sosnowiec Medical University of Silesia, 40-055 Katowice, Poland; ^3^Department of Biochemistry, Faculty of Medical Sciences in Zabrze, Medical University of Silesia, 41-808 Katowice, Poland; ^4^Department of Physiology, Faculty of Medical Sciences in Zabrze, Medical University of Silesia, 40-751 Katowice, Poland

## Abstract

Hucul and Arabian horses differ in the physiological constitution and exposition to environmental conditions. Oxidative stress plays a pathogenic role in many diseases and enables further injuries. The objective of this study was to compare the levels of enzymatic and nonenzymatic oxidative stress markers in Hucul horses living in seminatural conditions and in commercially handled Arabian horses. We tested the serum samples for total superoxide dismutase (total SOD), Cu-Zn-superoxide dismutase (CuZnSOD), and Mn-dependent superoxide dismutase (MnSOD) activity; for lipofuscin (LPS), ceruloplasmin (CER) and malondialdehyde (MDA) concentration; and for total antioxidant capacity (TAC) and total oxidant status (TOS). Total SOD (*p* < 0.001), MnSOD (*p* < 0.001), and CuZnSOD (*p* < 0.001) activities were significantly higher whereas LPS (*p* < 0.05), TAC (*p* < 0.001), TOS (*p* < 0.001), and MDA (*p* < 0.001) concentrations were significantly lower in the serum samples collected from Huculs vs. Arabian horses, regardless of the gender. Gender, regardless of the breed, had no significant impact on the antioxidants' activity and concentration. Total SOD and MnSOD activities were significantly higher in Hucul's mares when compared to Hucul's stallions. Concentrations of TAC and TOS were significantly lower in Arabian stallions than in Arabian mares. Commercially handled horses expressed a higher level of oxidative stress markers in comparison to breeds living in seminatural conditions. We conclude that antioxidants are important biomarkers of animal health, whether they are under maintenance care or performing physical exercise.

## 1. Introduction

Hucul and Arabian horses differ in the physiological constitution, conformation traits, exposition to environmental conditions, and nutritional requirements [1]. Huculs are one of the oldest primitive breeds of small mountain horse living in Eastern Europe. This pure horse breed is characterized by high physical endurance, high resistance to illnesses, low feed requirements, fertility, and longevity [2–4]. The Arabian horse breed is one of the oldest and the most influential pure horse breeds throughout the world [5]. Western Arabian populations selected in Poland and Europe originate from Middle Eastern Arabian populations' individuals imported around 200 years ago [5, 6].

Domestic animals are routinely exposed to a variety of anthropogenic stressors. Stress can be defined as physiological response elicited when a threat to homeostasis is perceived [7, 8]. Oxidative stress and reduction of the content of endogenous antioxidants in the body could increase the vulnerability of tissues and cellular components to oxygen reactive species [9, 10]. Oxidative stress occurs when oxidative substances and, particularly, reactive oxygen species overwhelm antioxidant defences. The balance between antioxidants and prooxidant compounds at a cellular level represents an important determinant of various physiological processes. Maintenance is the main aim of the so-called an integrated antioxidant system built in the animal body [11, 12]. Stress conditions and stress factors such as viral and parasite infections, intensive training, injury, and transportation may lead to an increased level of reactive oxygen species (ROS) in cells [7, 9, 11, 13, 14]. ROS are natural products of oxygen metabolism. Their cellular concentration is determined by the balance between production and clearance rates regulated by different antioxidant systems. Oxidative stress plays a pathogenic role in many diseases, leading to further injuries resulting from oxidative damage in differentiating cells [15]. However, together with growth factors and chemokines, ROS are influencing cellular regeneration and repair processes [14]. Cells' sensitivity to oxygen species is strictly controlled and modulated by ROS that are produced during metabolic processes and mainly used to eliminate pathogens and abnormally developing cells [16]. ROS are important in maintaining the cellular signal transduction, thus regulating cells' metabolism, homeostasis, defence systems, and adaptation processes [17, 18]. The antioxidant defence under the stress conditions consists of the cumulative activity of enzymatic and nonenzymatic antioxidant systems. Both enzymes and other antioxidant markers (e.g., total oxidant status (TOS), total antioxidant capacity (TAC), and ceruloplasmin) act like integrated parameters rather than the simple sum of measurable antioxidants [19]. Understanding the physiological processes behind oxidative stress is useful to efficiently identify the animals at risk of poor health and worse welfare outcomes. Horse breeds chosen for this study differ in the morphological constitution, traits, and disposition. They also differ in terms of physiological and biochemical variables [2, 20, 21]. Both breeds developed under severe but quite entirely different environmental conditions, characterized by a deficiency of food and heavy workloads. Thus, both breeds share selected common features, such as courage, strength, resistance to difficult habitat conditions, good health, and balanced character. Both breeds are characterized by good fodder utilization, fertility, and longevity [4]. The objective of this study was to analyse the levels of enzymatic and nonenzymatic antioxidant systems of adult individuals of two different breeds: Hucul horses, which live in seminatural conditions, in terms of human interventions, and the herds of Arabian horses that are commercially handled. To the best of our knowledge, there are no studies that analyse the nature of oxidative stress and levels of oxidative stress markers in Hucul and Arabian horses on the basic, physiological level.

## 2. Materials and Methods

**Figure 1:**
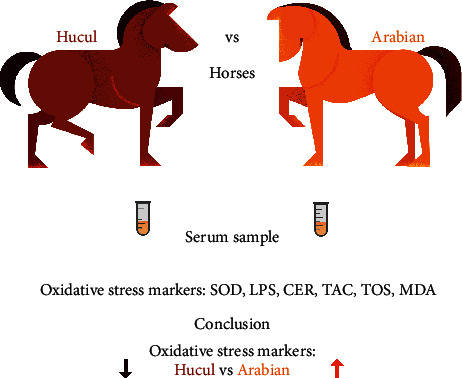
Experimental design of the study.

The study included 20 sexually mature, healthy Hucul horses (10 mares, 10 stallions) and 20 sexually mature, healthy Arabian horses (10 mares, 10 stallions). The Hucul horses aged between 5 and 10 years (7.5 ± 2.5), originated from a herd kept on extensive pasture (Poland) throughout the year and had no contact with other horses. Arabian horses, aged between 4 and 11 years (7.35 ± 3.65), originated from 20 different commercial horse riding clubs in Poland and were kept on the pasture. All horses were untrained and not transported for at least one month before collecting the samples. Female horses were not pregnant or in lactation. Both Arabian and Hucul horses were pure-breed mating. No history of diseases such as respiratory symptoms, injuries, digestive tract symptoms, signs from the central nervous system, or pregnancy complications were previously reported. Therefore, none of the horses showed any clinical signs of tested viral or microbiological infections at the time of sampling. All horses were therefore considered healthy based on history results of physical examination and haematological and biochemical analyses (data not shown). In this study, the blood samples were collected during routine veterinary care (Figure 1).

### 2.1. Blood Collection

Horse blood specimens submitted for routine veterinary towards equine viral arteritis (EVA) were used to assess oxidant stress markers. Therefore, the additional approval of the Local Ethics Committee was not required. A written consent statement was obtained from the owners of animals included in the study.

The blood samples were collected between 7:00 and 8:00 am during routine veterinary care. 10 ml of blood was collected from *Vena jugularis* to the vacuum tubes with EDTA. The samples were centrifuged at 4000 rpm for 10 min at 4°C. Then, the serum was collected and stored at –80°C until analysis.

### 2.2. Oxidative Stress Marker Analysis

Antioxidant systems in the Hucul and Arabian horse's serum samples were analysed by determining the enzymatic activity of total superoxide dismutase (total SOD), Cu-Zn-superoxide dismutase (CuZnSOD), and Mn-dependent superoxide dismutase (MnSOD); lipofuscin (LPS) and ceruloplasmin (CER) concentration; the total antioxidant capacity; (TAC) and total oxidant status (TOS). Lipid peroxidation rate was determined by measuring the malondialdehyde (MDA) concentration.

#### 2.2.1. Superoxide Dismutase (SOD) Analysis (EC 1.15.1.1)

The activity of SOD isoenzymes was measured using the Oyanagui method [22] with KCN as the inhibitor of the CuZnSOD isoenzyme in which the activity was calculated as the difference between total SOD activity and MnSOD activity. A blank sample containing ddH_2_O was used to calculate the total SOD activity. SOD activity is presented as nitrite units (NU) per ml serum. One NU exhibits 50% inhibition of the formation of a nitrite ion under the method's condition [22].

#### 2.2.2. Lipofuscin (LPS)

LPS concentration was determined according to Tsuchida et al. [23]. The serum was mixed with ethanol-ether (3 : 1 *v*/*v*), shaken and centrifuged. The intensity of fluorescence in a dissolved solid was determined using a PerkinElmer spectrophotometer LS45 (345 nm for absorbance, 430 nm for emission). The value is expressed in relative lipid extract fluorescence (RF), where 100 RF corresponds to the fluorescence of 0.1 *μ*g/ml quinidine sulfate in 0.1 N sulfuric acid. The inter- and intra-assay coefficients of variations (CV) were 2.8% and 9.7%, respectively.

#### 2.2.3. Ceruloplasmin (CER)

CER concentration in serum was assessed according to Richterich [24] using reaction with a p-phenyl diamine. Ceruloplasmin catalyzes the oxidation of colourless p-phenyl diamine replacing it with a blue-violet dye. The absorbance was read at 560 nm and the results are expressed in mg/ml. The measurement was conducted on a PerkinElmer VICTOR-X3 reader. Interassay and intra-assay coefficients of variations were, respectively, 1.3 and 4.0%.

#### 2.2.4. Total Antioxidant Capacity (TAC)

TAC of serum was measured using a commercial kit (Randox, Co. England). 2,2′-Azino-di-(3-ethylbenzothiazoline sulfonate) (ABTS) was incubated with a peroxidase (methmyoglobin) and hydrogen peroxide to produce the radical action ABTS+, which has a relatively stable blue-green colour that can be measured at 600 nm (PerkinElmer spectrophotometer LS45 for this study). The suppression of the colour was compared to the standard for TAC measurement assays (Trolox). The assay results are expressed as a Trolox equivalent (mmol/l). The inter- and intra-assay coefficients of variations (CV) were 1.1% and 3.8%, respectively.

#### 2.2.5. Total Oxidant Status (TOS)

The method uses the oxidation of Fe^2+^ to Fe^3+^ in an acidic medium. Fe^3+^ form a colourful complex with xylene orange ranging up to a blue-purple colouration. Absorption can be read at 560 nm wavelength. The measurements were done on VICTOR-X3 PerkinElmer reader. The concentration was calculated from the calibration curve using H_2_O_2_ as the standard. Values are expressed in *μ*mol/l as described by Erel [19].

#### 2.2.6. Lipid Peroxidation

Malondialdehyde (MDA) concentration in serum was determined using the reaction with thiobarbituric acid. The produced 1,1,3,3- tetraethoxypropane concentration can be read using the spectrophotometric method, employing 515 nm for excitation and 552 nm for emission using a PerkinElmer spectrophotometer LS45. MDA concentration was calculated from the standard curve prepared for 1,1,3,3-tetraethoxypropane [25].

### 2.3. Protein Concentration

Protein concentration was assessed according to the modified Lowry method [26, 27]. The assay was carried out by diluting the extracts to 1 ml with H_2_O and adding 0.9 ml of solution A (2 g l^−1^ potassium sodium tartrate (KNaC_4_H_4_O_6_·4H_2_O) and 100 g l^−1^ sodium carbonate (Na_2_CO_3_) in 0.5 M NaOH) before incubation for 10 min at 50°C. In the next step, the samples were cooled down to room temperature, and 1 ml of solution B (0.2 g l^−1^ KNaC_4_H_4_O_6_·4H_2_O and 0.1 g l^−1^ copper sulfate pentahydrate (CuSO_4_·5H_2_O) in 0.1 M NaOH) was added and left for 10 min. In the final step, 3 ml of solution C (Folin–Ciocalteu phenol reagent in H_2_O (1 : 16 *v*/*v*)) was added before incubation for 10 min at 50°C. A standard curve was made of bovine serum albumin (BSA; 0, 0.0625, 0.125, 0.25, 0.5, and 1 g l^−1^) and absorbance was read at 650 nm.

### 2.4. Statistical Analysis

Statistical analysis was performed using STATISTICA 12.5 PL (StatSoft, Poland). Statistical significance was set at a *p* value < 0.05. All tests were two tailed. Interval data were expressed as mean value ± standard deviation for normal distribution or as median/lower–upper quartile range for data with skewed or nonnormal distribution. Distribution of variables, including normality, was evaluated by the Shapiro-Wilk test and the quantile-quantile plot; homogeneity of variances was assessed by Levene's test. The two-way parametric ANOVA with post hoc contrast analysis, nonparametric Kruskal-Wallis test, or Mann–Whitney *U* test were used for data comparisons. The idea of the two-way ANOVA analysis is to compare two main factors and its interaction. In our case, the *p* value for race deals with the comparison between Hucul and Arabian horse independently to sex. Then, the second main factor deals with the comparison between gender (mare or stallion) independently to race. And finally, we get the interaction between those two main factors. When the two-way analysis of variance proved that one of the main analysed factors is statistically significant and when also, but not necessarily, the interaction between two main factors occurs, then contrast analysis may and should be conducted. It means that we may compare subclasses of the first factor between groups defined by the first factor, and also we may compare subclasses of the second factor between groups defined by the first factor (interaction). Those are contrast comparisons. They are defined as a priori in opposite to post hoc tests which compare all groups with each other (i.e., Tukey or Bonferroni tests). If at least one of the main factors was statistically significant, appropriate contrast analysis was performed and has been presented in Table 1. It is beyond our interest to check cross comparisons such as (3) Hucul mare vs. Arabian stallion and (4) Hucul stallion vs. Arabian mare, as they are biologically unjustified.

## 3. Results

**Figure 2:**
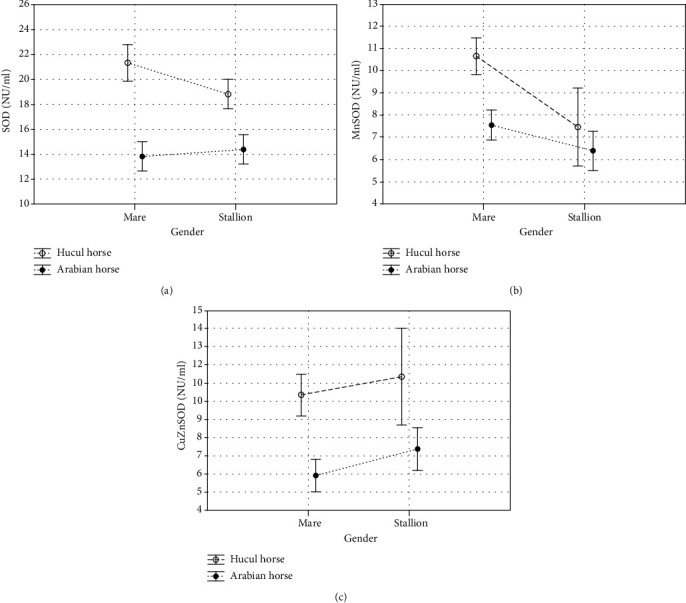
Antioxidant enzymatic activity in serum of Hucul and Arabian horses. (a) Mean total superoxide dismutase (SOD) activity (NU/ml). (b). Mean Mn-dependent superoxide dismutase (MnSOD) activity (NU/ml). (c) Mean Cu-Zn-superoxide dismutase CuZnSOD) activity (NU/ml). Vertical lines depict 95% confidence interval.

**Figure 3:**
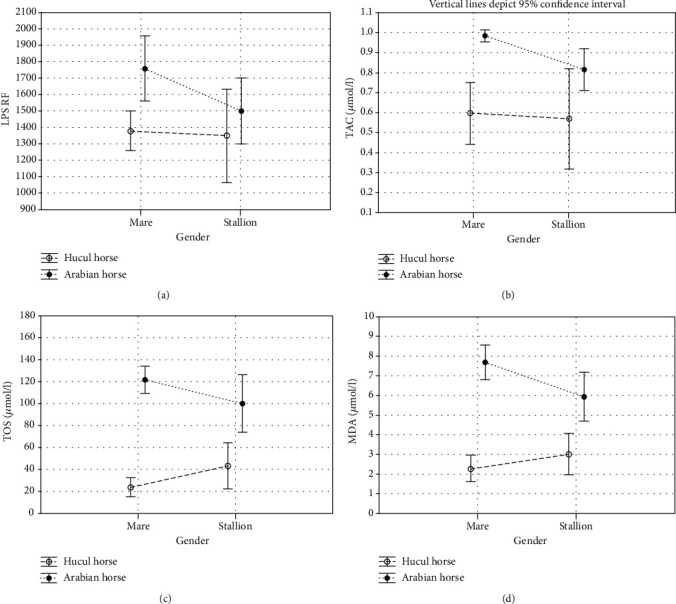
Antioxidant nonenzymatic markers in serum of Hucul and Arabian horses. (a) Mean lipofuscin relative fluorescence (LPS RF). (b) Mean total antioxidant status (TAC) (*μ*mol/l). (c) Mean total oxidative status (TOS) (*μ*mol/L). (d) Mean malondialdehyde (MDA) concentration (*μ*mol/g). Vertical lines depict 95% confidence interval.

**Figure 4:**
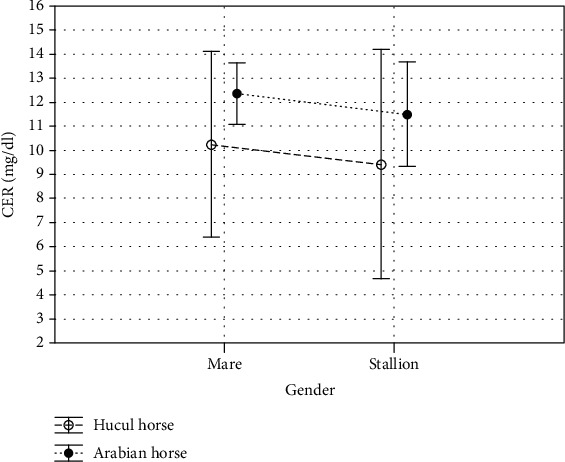
Mean ceruloplasmin (CER) concentration (mg/dl) in serum of Hucul and Arabian horses. Vertical lines depict 95% confidence interval.

The results of antioxidant enzymatic activity and nonenzymatic antioxidants concentrations in the blood serum of Hucul and Arabian horses are presented in Table 1 and Figures 2–4.

In the presented study, we observed breed-dependent differences in the antioxidant enzymatic activity and nonenzymatic antioxidants concentration. Total SOD, MnSOD, and CuZnSOD activities were significantly higher (*p* < 0.001, Figures 2(a)–2(c); Table 1) whereas the concentrations of nonenzymatic markers such as LPS, TAC, TOS, and MDA were significantly lower (*p* < 0.05, *p* < 0.001, respectively, Figures 3(a)–3(d); Table 1) in the serum samples collected from Huculs when compared to serum samples collected from Arabian horses regardless of the gender. The concentration of ceruloplasmin was at the same level in all analysed study groups (*p* < 0.097, Figure 4, Table 1).

Intergroup analysis shows that gender, regardless of the breed, had no significant impact on the antioxidants' activity and concentration in both analysed breed groups except for MnSOD activity (*p* < 0.001, Table 1).

Analysis of interaction between breed and gender shows that those factors combined influenced SOD and MnSOD activities (*p* < 0.05 and *p* < 0.001, respectively, Table 1) as well as TOS and MDA concentration (*p* < 0.001, Table 1).

Hucul's gender intragroup analysis showed that SOD and MnSOD activities were significantly higher in mares when compared to stallions (*p* < 0.05, *p* < 0.001, respectively, Table 1). Arabian gender intragroup analysis showed that nonenzymatic antioxidants concentrations of TAC (*p* < 0.05), TOS (*p* < 0.05), and MDA (*p* < 0.01) were significantly lower in stallions than in mares (Table 1).

The presented intragroup comparison showed that, in relation to gender, activities of antioxidant enzymes are significantly higher while concentrations of nonenzymatic antioxidants are lower in Hucul mares when compared to Arabian mares, in terms of all analysed parameters (Table 1). SOD and ZnCuSOD activities were significantly higher in Arabian stallions when compared to Huculs' stallions (*p* < 0.001 for both parameters, Table 1), whereas concentration of TAC, TOS, and MDA were significantly higher in Arabian stallions when compared to Huculs' stallions (*p* < 0.001, for all three parameters, Table 1).

## 4. Discussion

In this study, we report that commercially handled Arabian horses showed a higher level of oxidative stress, measured with antioxidant enzymatic activity (SOD), accumulation of oxidative stress products (LPS and MDA), and total oxidant status (TOS), than Hucul horses.

In this work, we observed that total SOD, MnSOD, and CuZnSOD activities were significantly higher in blood samples obtained from Huculs when compared to Arabian horses. The superoxide dismutase family (SODs) is the first line of defence against reactive oxygen species (ROS) [28]. It is known, that changes in SOD activity protect a host against microorganisms [29, 30], bacteria [31, 32], and parasites [31] and those changes depend on physical activity, diet, and chemical factors [33, 34]. A lower level of total SOD activity in Arabian horses may result from the depletion of the antioxidant defence system due to long exposure to oxidative stress caused by vaccination, handling, and commercial usage. Mitochondria, as the main oxygen-metabolizing organelles of the cell, are responsible for intensive ROS production [35, 36]. Manganese superoxide dismutase (MnSOD) is one of the main ROS-neutralizing enzymes, because of its localization in mitochondria. Increased activity or expression of MnSOD can significantly influence mitochondrial function, stimulating the development of different diseases [37, 38]. Manganese superoxide dismutase (MnSOD) controls the levels of O_2_^·−^ in mitochondria. A product of MnSOD-catalyzed reactions is hydrogen peroxide (H_2_O_2_), thus increased MnSOD activity results in H_2_O_2_ accumulation [39]. The increase in protective MnSOD protein level observed for Huculs in this study may explain, at least in part, the improvement of TAS in Hucul horses when compared to Arabian horses.

Lipofuscin is an undegradable material, composed of oxidatively modified protein and lipid residues. Its accumulation is proportional to changes observed in mitochondria and depends on individual's age and ROS formation intensity [40]. MDA is widely used as a biochemical marker of peroxidative damage to cell membranes induced by physical and/or chemical oxidative stress [41]. Ceruloplasmin is an antioxidant protein (a2-serum glycoprotein) which transports 95% of copper in blood [42, 43]. The levels of oxidant species can be analysed separately, but separate assessments are time consuming, expensive, and require complicated techniques [19]. While the oxidant effects are additive, the tissue oxidant/antioxidant status may be assessed by measuring the total oxide status (TOS); therefore, TOS levels may be described as the main indicator of oxidant molecules [19]. TAC is considered the cumulative activity of all the antioxidants present in the cells, strongly influenced by oxidative stress (OS) [43–45]. Thus, TOS and TAC are relevant indicators of the oxidative and antioxidative status. Here, nonenzymatic antioxidant systems represented by LPS, TAC, TOS, and MDA were significantly lower in Huculs when compared to Arabian horses, regardless of gender. Higher level of SOD activity and lower lipids' peroxidation rate, as measured by MDA concentration, show that Huculs antioxidant systems are more efficient than Arabian horses or the nature of stressors is relatively different. The significantly higher level of MDA serum concentration of Arabian horses corresponds with results of the study on MDA evaluated in patients' serum [46]. We found that TAS was significantly higher in Arabian horses, which suggests that this breed shows higher oxidative damage, as well as different antioxidant capacity than Hucul horses. That may result from all types of the stress connected to handling, environmental exposure, and nutritional requirements.

Here, we observed that Arabian mares showed a higher level of oxidative stress, measured by increased MDA levels, higher mobilisation of cellular antioxidant agents (TAC), and elevated TOS, in comparison with Arabian stallions. That may suggest that Arabian mares, due to gender and constitution, are more sensitive, so their physiological answer to the environmental stressors is stronger than in Arabian stallions. It may also result from the stress connected to handling, environmental exposition, and nutritional requirements. On the contrary, the reaction to the oxidative stress in Hucul mares and Hucul stallions is at a comparable level except for the higher total SOD and MnSOD antioxidant activities observed for Hucul mares. This subtle mobilisation of SOD enzymes could result from gender differences in sensibility to the general stress conditions.

The intergroup comparison shows that Huculs' antioxidant systems are more efficient than Arabian ones. Huculs are known for their excellent health, including disease resistance, tolerance for environmental conditions, and longevity [47]. The analysed population of Huculs lives in hordes as a primitive breed, in conditions that are close to their natural habitat, spending all their lives on pasture. The pattern of antioxidant systems shows the natural capability of Hucul herds to adapt to the natural stressors, even without any medical intervention. In opposite, hot-blooded Arabian horses used for endurance riding, exposed to intensive learning and training conditions, kept in close contact with humans—thus far from their natural environment—show higher level of response to oxidative stress.

## 5. Conclusions

Horse breeds chosen for this study differ in the morphological constitution, traits, and disposition. They also differ in terms of physiological and biochemical variables. Both breeds developed under severe environmental conditions, characterized by a deficiency of food and heavy workloads. Thus, both breeds share numerous common features, such as courage, strength, and resistance to difficult habitat conditions; good health; and balanced character. Antioxidant stress markers are important biomarkers of animal conditions, in this case, referring to the adaptation of the physiological processes under homeostatic conditions. Based on the results obtained in this study, we can conclude that the physiological level of oxidative/antioxidative defence measured with profiles of antioxidant stress markers was higher in commercially handled Arabian horses than in semi-isolated Hucul horses.

## Figures and Tables

**Table 1:** Comparison of enzymatic and nonenzymatic antioxidant systems in the blood serum of Hucul and Arabian horses. Descriptive statistics and results of two-way analysis of variance. Mean ± standard deviation or median (lower–upper quartile). Statistical significance was set at a *p* < 0.05.

	Hucul horses		Arabian horses		*p*_breed_	*p*_gender_	*p*_interaction_	*p*_1_	*p*_2_	*p*_3_	*p*_4_
	Mare	Stallion	Mare	Stallion							
Total SOD (NU/ml)	21.34 ± 2.06	18.81 ± 1.29	13.82 ± 2.42	14.38 ± 1.46	<0.001	0.145	<0.05	<0.05	0.523	<0.001	<0.001
MnSOD (NU/ml)	10.66 ± 1.17	7.46 ± 1.90	7.54 ± 1.41	6.39 ± 1.05	<0.001	<0.001	<0.05	<0.001	0.055	<0.001	0.145
CuZnSOD (NU/ml)	10.35 ± 1.58	11.35 ± 2.84	5.90 ± 1.83	7.37 ± 1.42	<0.001	0.054	0.707	0.296	0.076	<0.001	<0.001
LPS (RF)	1379.85 ± 166.66	1347.06 ± 306.46	1757.69 ± 411.18	1499.47 ± 239.94	<0.05	0.176	0.293	0.839	0.068	<0.01	0.372
CER (mg/dl)	10.24 ± 5.37	9.43 ± 5.13	12.36 ± 2.60	11.51 ± 2.59	0.097	0.507	0.984	—	—	—	—
TAC (mmol/l)	0.60 ± 0.22	0.57 ± 0.27	0.98 ± 0.06	0.82 ± 0.12	<0.001	0.069	0.191	0.724	<0.05	<0.001	< 0.001
TOS (*μ*mol/l)	23.72 ± 12.19	43.28 ± 22.75	121.76 ± 25.65	100.13 ± 31.46	<0.001	0.895	<0.05	0.107	<0.05	<0.001	<0.001
MDA (*μ*mol/l)	2.28 ± 0.94	3.03 ± 1.13	7.69 ± 1.80	5.94 ± 1.48	<0.001	0.307	<0.05	0.315	<0.01	<0.001	<0.001

Abbreviations: total SOD: superoxide dismutase; LPS: lipofuscin; CER: ceruloplasmin; TAC: total antioxidant capacity; TOS: total antioxidant status; MDA: malondialdehyde. *p*_breed_: effect of breed; *p*_gender_: effect of gender; *p*_interaction_; interaction between gender and breed; *p*_1_: Hucul mare vs. Hucul stallion; *p*_2_: Arabian mare vs. Arabian stallion; *p*_3_: Hucul mare vs. Arabian mare; *p*_4_: Hucul stallion vs. Arabian stallion.

## Data Availability

The original data are available after contact with corresponding author.
